# Lipid Mixtures Containing a Very High Proportion of Saturated Fatty Acids Only Modestly Impair Insulin Signaling in Cultured Muscle Cells

**DOI:** 10.1371/journal.pone.0120871

**Published:** 2015-03-20

**Authors:** Sean A. Newsom, Allison C. Everett, Sanghee Park, Douglas W. Van Pelt, Alexander Hinko, Jeffrey F. Horowitz

**Affiliations:** Substrate Metabolism Laboratory, School of Kinesiology, University of Michigan, Ann Arbor, Michigan, United States of America; University of Birmingham, UNITED KINGDOM

## Abstract

*In vitro* examinations of the effect of saturated fatty acids on skeletal muscle insulin action often use only one or two different fatty acid species, which does not resemble the human plasma fatty acid profile. We compared graded concentrations (0.1-0.8mM) of 3 different lipid mixtures: 1) a physiologic fatty acid mixture (NORM; 40% saturated fatty acids), 2) a physiologic mixture high in saturated fatty acids (HSFA; 60% saturated fatty acids), and 3) 100% palmitate (PALM) on insulin signaling and fatty acid partitioning into triacylglycerol (TAG) and diacylglycerol (DAG) in cultured muscle cells. As expected, PALM readily impaired insulin-stimulated pAkt^Thr308^/Akt and markedly increased intracellular DAG content. In contrast, the fatty acid mixtures only modestly impaired insulin-stimulated pAkt^Thr308M^/Akt, and we found no differences between NORM and HSFA. Importantly, NORM and HSFA did not increase DAG content, but instead dose-dependently increased TAG accumulation. Therefore, the robust impairment in insulin signaling found with palmitate exposure was attenuated with physiologic mixtures of fatty acids, even with a very high proportion of saturated fatty acids. This may be explained in part by selective partitioning of fatty acids into neutral lipid (i.e., TAG) when muscle cells were exposed to physiologic lipid mixtures.

## Introduction

Epidemiological studies provide considerable support for the notion that diets high in saturated fats are associated with accelerated development of several cardiometabolic abnormalities, including insulin resistance and type 2 diabetes [1]. To investigate mechanisms underlying these associations, experiments performed *in vitro* commonly incubate cultured cells in high concentrations of a single saturated fatty acid (e.g. palmitate (C16:0)), which potently impairs insulin signaling [2–4]. Interestingly, the addition of an unsaturated fatty acid (e.g. oleate (18:1)) to palmitate in the incubation media can attenuate, and even completely prevent the deleterious effect(s) of palmitate on insulin signaling in cultured muscle cells [5–8]. However, exposing muscle cells to media containing only one or even two different fatty acids (often in non-physiologic concentrations/proportions) is not likely to provide an accurate reflection of elevated fatty acid availability common in obesity [9–12]. Thus, the primary aim of this study was to determine the effects of physiologic lipid mixtures (resembling the human plasma fatty acid profile) containing a normal *vs*. high proportion of saturated fatty acids on insulin signaling in cultured muscle cells.

Impaired insulin action in obesity is linked to an excessive fatty acid uptake into insulin sensitive tissues, including skeletal muscle [13]. Skeletal muscle accumulation of bioactive lipids such as diacylglycerol (DAG) is associated with the development of insulin resistance [14–16]. However, because preferentially incorporating fatty acids into triacylglycerol (TAG) is considered protective (as it limits fatty acid substrate available for the formation of more bioactive lipid intermediates) [17], a secondary aim was to determine the effects of these lipid mixtures on muscle cell fatty acid partitioning into DAG and TAG.

## Materials and Methods

### Cell culture

Mouse C2C12 myoblasts (American Type Culture Collection, Manassas, VA) were grown in high glucose Dulbecco’s Modified Eagle Medium (DMEM) supplemented with 10% (v/v) fetal bovine serum and 1% (v/v) antibiotic-antimycotic solution in tissue culture treated plates. All cells were passed 3–5 times, and ultimately plated in 6-well, 35mM/well tissue culture treated plates. Upon reaching ~70% confluence, myoblasts were switched to high glucose (~25mM) DMEM supplemented with 2% (v/v) horse serum and 1% (v/v) antibiotic-antimycotic solution to induce differentiation. This media was replaced at 48 h. At 96 h (4 d), differentiated myotubes were used in the experiments described below. All cell culture media and media supplements were purchased from Gibco-Invitrogen (Grand Island, NY).

### Experimental procedures

Differentiated mouse C2C12 myotubes were incubated for 12 hours in media containing low glucose (~5mM) serum-free DMEM (with 1% (v/v) antibiotic-antimycotic solution), and 2% (w/v) fatty acid-free bovine serum albumin (BSA) supplemented with one of three different fatty acid mixtures: 1) a normal physiologic mixture of fatty acids reflecting their proportion in plasma of a healthy human (**NORM**; 30% oleate [C18:1], 25% linoleate [C18:2], 25% palmitate [C16:0], 15% stearate [C18:0], and 5% palmitoleate [C16:1], thus 40% saturated fatty acids), 2) a physiologic mixture of fatty acids resembling a diet very high in saturated fatty acids (**HSFA**; 20% oleate, 15% linoleate, 35% palmitate, 25% stearate, and 5% palmitoleate, thus 60% saturated fatty acids), or 3) 100% palmitate (**PALM**). The NORM and HSFA fatty acid mixtures were formulated by Nu-Chek Prep Inc. (Elysian, MN). We performed incubations at 4 different concentrations of the 3 fatty acid mixtures (0.1, 0.2, 0.4, or 0.8mM), and a “control” treatment, without fatty acid (0mM). After the 12 hour incubation, myotubes were treated without or with insulin (100nM) for 15min, and then harvested for later analysis (described below). All measures of lipid accumulation and lipid metabolism regulatory proteins were made using non-insulin treated myotubes. All analyses were performed on n≥3 experiments.

### Ethics statement

Written, informed consent was obtained from all human subjects prior to participation in the studies described below. Importantly, all procedures were approved by the University of Michigan Institutional Review Board, in accordance the principals expressed in the Declaration of Helsinki.

### Human primary muscle cell culture

Some experiments were repeated in primary myotubes cultured from human skeletal muscle (n≥3 experiments). Vastus lateralis biopsies were obtained from 6 obese non-diabetic men and women (male/female: 1/5; age 32±2years; BMI 39.0±1.7kg/m^2^). Muscle biopsy samples (30–50mg) were dissected free of adipose and connective tissue, rinsed in saline, and minced in low glucose DMEM (with 1% (v/v) antibiotic-antimycotic solution). Minced muscle was digested at 37°C in low glucose DMEM supplemented with 0.5% type II collagenase. Digested muscle was resuspended in low glucose growth media and placed in untreated tissue culture plates at 37°C to facilitate adherence of non-satellite cell populations. Non-adhering supernatant (containing satellite cells) was filtered (100μm), resuspended in low glucose growth media, and placed in a Matrigel-treated plate (Sigma-Aldrich). Experiments were performed following 5–7d of differentiation as described for C2C12 muscle cells.

### Cell harvest

Cells were rinsed twice with ice-cold Dulbecco’s phosphate buffered saline (DPBS), treated with lysis buffer (20mM Tris-HCl pH 7.5, 150mM NaCl, 1mM Na_2_EDTA pH 8.0, 1mM EGTA pH 8.0, 1% (v/v) Triton X-100, 2.5mM NaPP, 1mM β-glycerophosphate, 1mM Na_3_VO_4_, and 1x SigmaFAST protease inhibitor cocktail), and scraped on ice into microfuge tubes. Lysates were centrifuged at 20,000 g for 10 min at 4°C. Supernatants were collected and tested for protein concentration (Pierce BCA protein assay, Thermo Scientific, Rockford, IL). For cell lipid content assays (described below), DPBS was used as a harvest solution and lysates were not centrifuged prior to use in cell lipid content assays.

### Western blotting

20–30 μg of protein were separated by SDS-PAGE and transferred to nitrocellulose membranes. To minimize potential variability between analyses, all western blots were performed using aliquots of the same master preparation for each sample. Furthermore, to help ensure equal loading and proper transfer of proteins, all membranes were visually inspected using Ponceau-S staining immediately after transfer. Various proteins were targeted within whole cell lysates with primary antibodies against: Akt (9272; Cell Signaling Technology, Danvers, MA), pAkt^Thr308^ (9275; Cell Signaling Technology), GSK3β (9315; Cell Signaling Technology), pGSK3α/β^Ser21/9^ (9331; Cell Signaling Technology), AS160 (ABS54; EMD Millipore, Billerica, MA), pAS160^Thr642^ (3028 P1; Symansis, Auckland, New Zealand), GPAT1 (4613; ProSci Incorporated, Poway, CA), DGAT1 (NB110–41487; Novus Biologicals, Littleton, CO), ATGL (2138; Cell Signaling Technology), CGI-58 (NB110–41576; Novus Biologicals), and HSL (4107; Cell Signaling Technology). Membranes were incubated with appropriate secondary antibodies and developed using enhanced chemiluminescence (Amersham Biosciences, Piscataway, NJ). Bands were imaged and then quantified via densitometry (AlphaEaseFC, Alpha Innotech Corp., Santa Clara, CA). Data are presented in arbitrary units relative to values obtained for 0mM (control) treated myotubes.

### Cellular triacylglycerol and diacylglycerol concentrations

Cells were harvested in ice-cold DPBS, and lipids were extracted overnight at 4°C in a single-phase mixture of chloroform-methanol-aqueous homogenate (1:2:0.8, v/v/v). Internal lipid markers for TAG, DAG, monoacylglycerol, non-esterified fatty acid (NEFA), phospholipid (PL), and cholesterol ester having fatty acid moieties of odd carbon number were added at the start of extraction for subsequent purity and recovery determinations (Nu-Chek Prep Inc., Waterville, MN; Avanti Polar Lipids Inc., Alabaster, AL). Fatty acid methyl esters from TAG and DAG were generated, purified, and detected as previously described [18]. Data are presented in arbitrary units relative to values obtained for 0mM (control) treated myotubes.

### Statistical analysis

A two-way (*dose x treatment type*) or one-way (*treatment type*) ANOVA was used to test for significant differences in factor means. Tukey’s post-hoc pair-wise analysis was used to examine significant *F* values. Statistical significance was defined as *P*≤0.05.

## Results

### Insulin signaling

**Fig 1:**
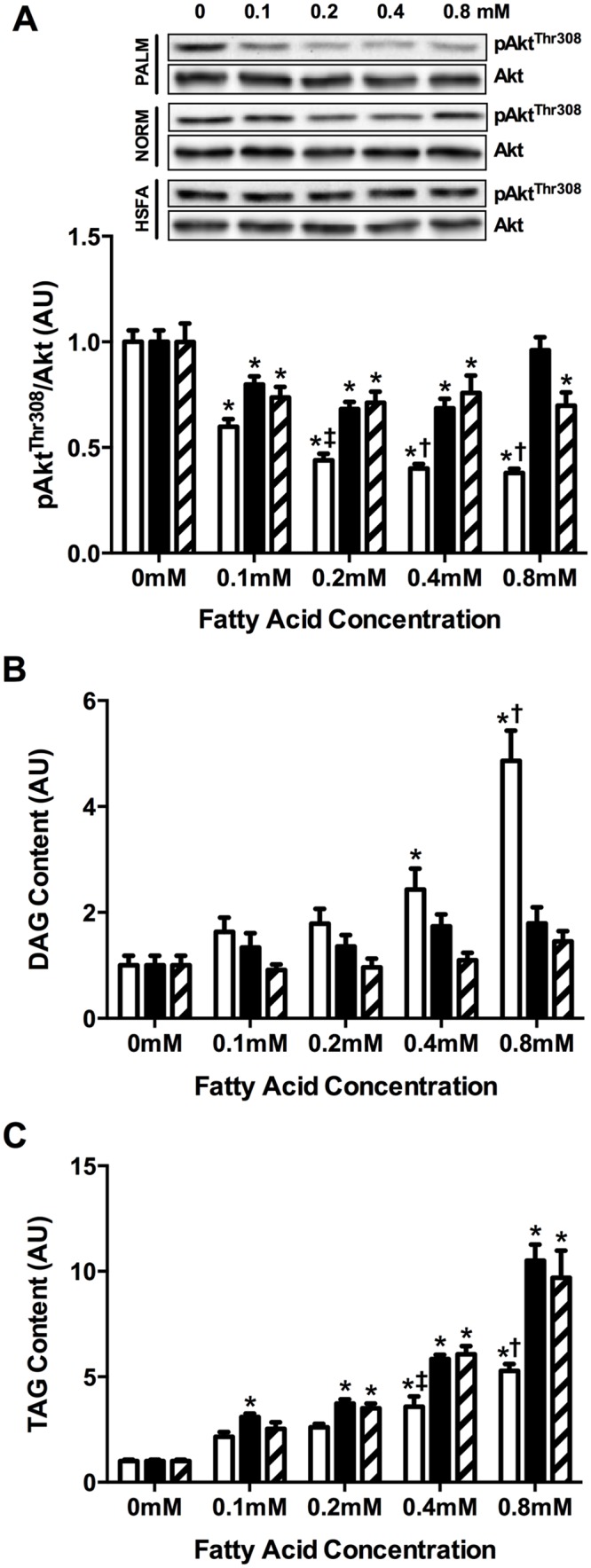
Insulin signaling and fatty acid partitioning in C2C12 muscle cells. Muscle cells were incubated with PALM (open), NORM (filled), or HSFA (hatched). (A) Insulin-stimulated pAkt^Thr308^/Akt, and non-insulin-stimulated (i.e., basal) accumulation of (B) DAG and (C) TAG. All data are expressed relative to a no fatty acid (0mM) condition, and representative blots are inset above figure panel A. *P<0.05 *vs*. 0mM. †*P*<0.05 *vs*. NORM and HSFA within treatment dose. ‡*P*<0.05 *vs*. HSFA within treatment dose. DAG, diacylglycerol; TAG, triacylglycerol; AU, arbitrary units.

As anticipated, incubating the myotubes in PALM readily suppressed insulin-stimulated pAkt^Thr308^/Akt even at the lowest dose (0.1mM; *P*<0.001), and this effect was dose-dependent (Fig. 1A). In contrast, incubating myotubes in a mixture of fatty acids resembling a “normal” plasma fatty acid profile (NORM), induced a lesser (yet statistically significant) suppression in insulin-stimulated pAkt^Thr308^/Akt (Fig. 1A). Interestingly, incubating myotubes in the fatty acid mixture containing a very high physiologic proportion of saturated fatty acids (HSFA) did not exacerbate the modest suppression in insulin-stimulated pAkt^Thr308^/Akt observed with NORM. Consequently, PALM treatment resulted in significantly lower insulin-stimulated pAkt^Thr308^/Akt compared with NORM and HSFA at the 0.4 and 0.8mM doses, but there was no difference between NORM and HSFA (Fig. 1A). Importantly, basal (i.e. non-insulin stimulated) phosphorylation of Akt was not altered by exposure to any of the various treatments, at any treatment dose (S1A Fig).

Despite the marked reduction in pAkt^Thr308^/Akt in response to PALM incubation, this was not accompanied by a similarly robust suppression in insulin-stimulated phosphorylation of targets downstream of Akt, including pGSK3β^Ser9^/GSK3β and pAS160^Thr642^/AS160 (S1B and S1C Fig). This phenomenon may be explained by the fact that insulin induced only a mild increase in the phosphorylation of both GSK3β and AS160 (~50% and ~100% increase, respectively, compared with >10-fold increase in pAkt^Thr308^), making it challenging to detect a reduction in phosphorylation in response to PALM.

### Fatty acid partitioning

PALM incubation increased cellular DAG content in a dose-dependent manner (Fig. 1B). Conversely, neither NORM nor HSFA increased DAG (Fig. 1B). Importantly, the pattern of change in cellular TAG content in response to the different treatments was nearly opposite to that of DAG (Fig. 1C). Both NORM and HSFA resulted in robust, dose-dependent increases in cellular TAG content, while this effect was significantly attenuated with PALM (Fig. 1C). In general, the fatty acid composition of both DAG and TAG tended to resemble the fatty acid(s) provided in the incubation media, particularly at the higher treatment doses when lipid accumulation was greatest (S1 and S2 Tables).

### Factors regulating lipid storage and breakdown

**Fig 2:**
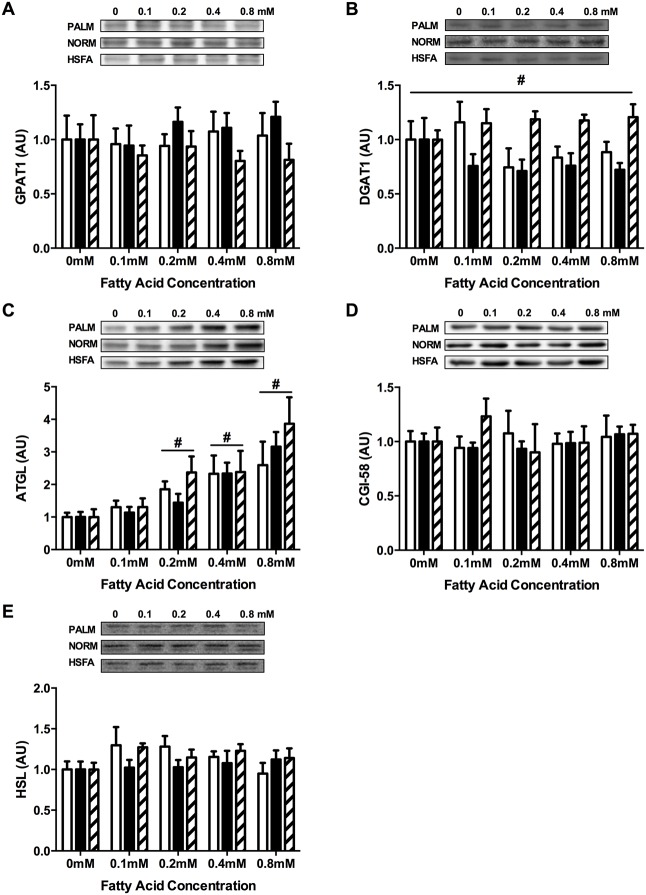
Factors regulating lipid storage and breakdown in C2C12 muscle cells. Muscle cells were incubated with PALM (open), NORM (filled), or HSFA (hatched). Protein abundance of (A) GPAT1, (B) DGAT1, (C) ATGL, (D) CGI-58, and (E) HSL. In all figure panels data are expressed relative to a no fatty acid control condition (0mM). In panel B, #*P*<0.05 for a main effect of HSFA *vs*. PALM and NORM. In panel C, #*P*<0.05 for a main effect of treatment dose *vs*. 0mM. Representative blots are inset above each figure panel. GPAT, glycerol-3-phosphate acyltransferase; DGAT, diacylglycerol acyltransferase; ATGL, adipose triacylglycerol lipase; CGI-58, comparative gene identification 58; HSL, hormone sensitive lipase; AU, arbitrary units.

The fatty acid partitioning pattern in PALM compared with NORM and HSFA treated muscle cells was not explained by differences in protein abundance of key TAG synthesis enzymes GPAT1 and DGAT1, or lipolytic regulators, including ATGL co-activator CGI-58 and HSL (Fig. 2). It is noteworthy that protein abundance of the TAG lipase ATGL was increased dose-dependently in all fatty acid treatments (Fig. 2C). However, this effect seems unlikely to have been a major determinant of fatty acid partitioning.

### Human primary muscle cell culture

**Fig 3:**
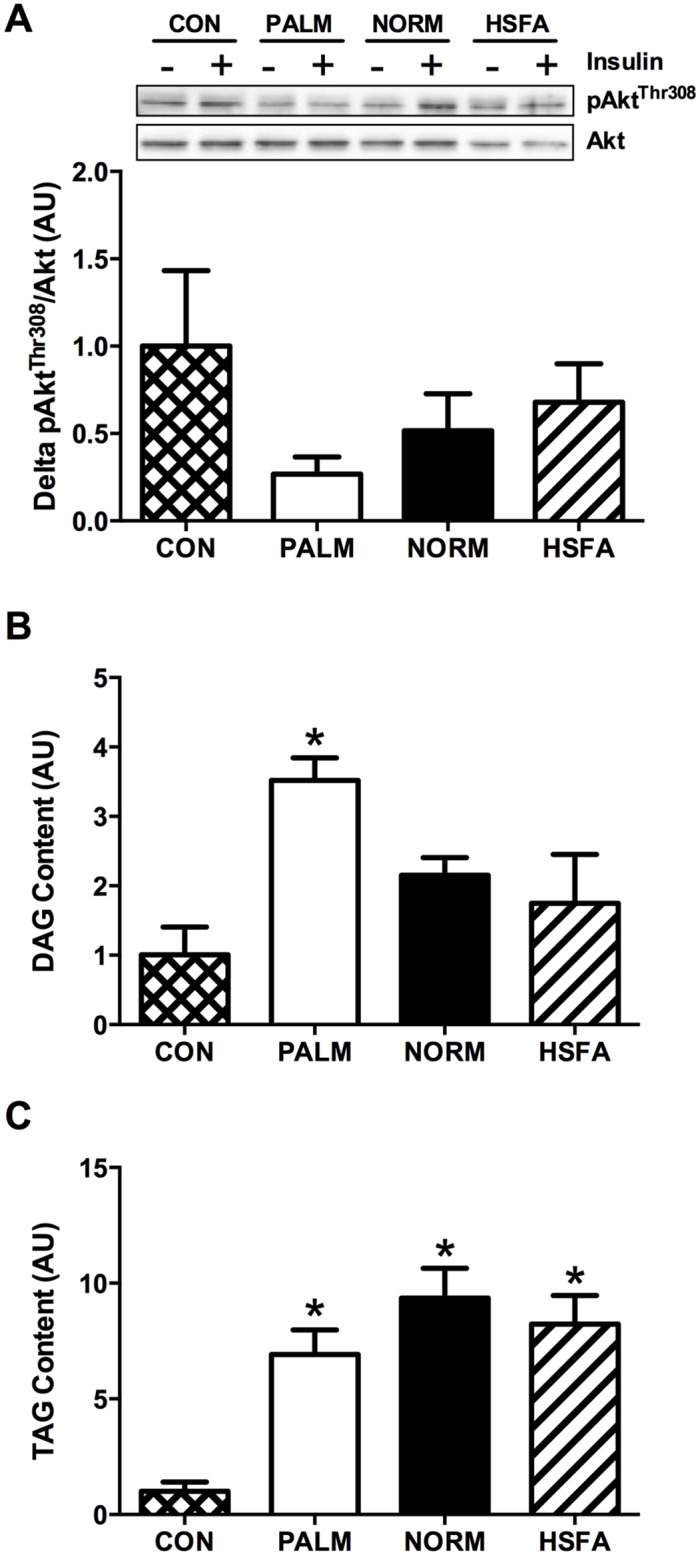
Insulin signaling and fatty acid partitioning in human primary skeletal muscle cells. Muscle cells were incubated with vehicle (CON; cross-hatched), PALM (open), NORM (filled), or HSFA (hatched). (A) Delta pAkt^Thr308^/Akt (calculated as the difference between insulin-stimulated and basal pAkt^Thr308^/Akt), and non-insulin-stimulated (i.e., basal) accumulation of (B) DAG and (C) TAG. Cells were exposed for 12h to a no fatty acid control condition (CON) or 0.4mM of one of the 3 different fatty acid treatments (PALM, NORM, and HSFA). For all panels, data are expressed relative to CON. *P<0.05 *vs*. CON. DAG, diacylglycerol; TAG, triacylglycerol; AU, arbitrary units.

Unlike the C2C12 experiments, human primary muscle cells tended to exhibit modest differences in basal pAkt^Thr308^/Akt in response to fatty acid treatment. To account for this, insulin-stimulated pAkt^Thr308^/Akt was determined as the increase above basal pAkt^Thr308^/Akt (i.e. delta pAkt^Thr308^/Akt). The effects of our fatty acid treatments on the suppression of insulin-stimulated pAkt^Thr308^/Akt in the human primary cells (Fig. 3A), resembled our findings in C2C12 myotubes (Fig. 1A), but none of these differences in our human cells reached statistical significance. Fatty acid partitioning within the human primary muscle cells reinforced our observations from C2C12 experiments; PALM primarily increased DAG concentration, while all lipid treatments increased TAG content (Fig. 3B and 3C).

## Discussion

The major finding of this study was that unlike the robust suppression in insulin signaling found when cultured myotubes were incubated in palmitate, exposing myotubes to a physiologic mixture of fatty acids containing a very high proportion of saturated fatty acids (60%) only modestly impaired insulin-stimulated phosphorylation of Akt. Moreover, insulin signaling in response to this high saturated fatty acid mixture (HSFA) was no different to that found when cultured myotubes were incubated in a fatty acid mixture with a relatively “normal” proportion of saturated fatty acids (NORM; 40% saturated fatty acids). These findings suggest that when muscle cells are exposed to the most abundant fatty acids in human plasma, in proportions and at concentrations generally found in humans, the saturation state of the fatty acids may not be a critical factor regulating insulin signaling.

We acknowledge that this does not agree many epidemiological reports supporting the notion that diets high in saturated fats are associated with insulin resistance and an increased prevalence of type 2 diabetes [1]. However, well-controlled dietary intervention studies in human subjects have also reported that diets high in saturated fatty acids do not induced marked insulin resistance [19]. Still, the reason for this discrepancy between the epidemiological studies and intervention studies (as well as *in vitro* studies like ours) is not clear. It is possible that epidemiological associations between the type of dietary fat and insulin resistance are mediated by other unappreciated dietary and/or environmental conditions that may complement the saturation state of dietary fat. Conversely, it is also possible that some intervention studies did not expose participants to the diet for sufficient duration to evoke changes in insulin action. Along these lines, it is noteworthy that although we incubated the muscle cells in our lipid mixtures for several hours, in human obesity muscles are *chronically* exposed to the fatty acid milieu available in the systemic circulation [9–11]. It is also possible that the deleterious effects of a highly saturated fatty acid profile in humans may be predominately mediated via impairments in other tissues (e.g. adipose, liver, vascular) and/or secondary to resulting proinflammatory/stress response(s) in these other tissues.

Similar to previous studies [4, 5], we found incubating muscle cells in palmitate induced a robust accumulation of DAG. In contrast, neither of our physiologic fatty acid mixtures increased DAG concentration, but instead increased TAG accumulation in a dose-dependent manner. In fact, accumulation of DAG and TAG in response to NORM and HSFA was nearly identical despite a substantial difference in the contribution of saturated fatty acids to the two physiologic fatty acid mixtures (i.e. 40% *vs*. 60%, respectively). The tendency for the fatty acid moieties within each lipid pool (both DAG *and* TAG) to reflect the fatty acid composition of the incubation media indicates relatively unbiased incorporation of fatty acids into these lipid pools. Akin to our insulin signaling observations, these data suggest that when skeletal muscle cells are exposed to a physiologic mixture of fatty acids, the saturation state of these fatty acids is not a critical factor regulating their partitioning between DAG and TAG.

At present it is unclear why incubating skeletal muscle cells with physiologic mixtures of fatty acids results in robust accumulation of TAG, whereas incubation with 100% palmitate is associated with significant DAG accumulation. The protein abundance of key lipogenic and lipolytic enzymes measured here does not appear to have played a significant role in this regard [17, 20]. Previous work in INS-1 cells and isolated mouse islets reported similarly attenuated palmitate- compared with oleate-mediated TAG accumulation *in vitro* [21, 22], and the authors suggested these effects may be due to the non-physiologic accumulation of tripalmitin [22]. Along these lines, the non-physiologic cytotoxicity of 100% palmitate exposure in cultured muscle cells has also been well documented [23]. Regardless of mechanism(s), because intramyocellular DAG has been linked to impaired insulin signaling, the preferential storage as TAG may at least partly explain why exposure to high concentrations of our physiologic lipid mixtures resulted in only modest suppression of insulin-stimulated phosphorylation of Akt. These findings support the working hypothesis that the capacity to esterify and store fatty acids as TAG may help limit the accumulation of more harmful lipid intermediates, such as DAG, and thereby “protect” against fatty acid-induced insulin resistance [17, 24, 25]. Nevertheless, regulation of intramyocellular fatty acid partitioning remains an unresolved process worthy of continued investigation.

One limitation of this investigation is that a constant concentration of BSA was provided for every fatty acid incubation (and control) condition. As such, the molar ratio of fatty acid to BSA increased with increasing fatty acid concentration in the media, resulting in a sub-optimal molar ratio during the higher fatty acid treatments [12]. Despite this, it is important to recognize that this cannot account for the differences observed between our physiologic mixtures and palmitate, because within each treatment dose (i.e. 0.1, 0.2, 0.4, 0.8mM) the molar ratio of fatty acid to BSA was the same among the different incubations. Additionally, experiments using human primary skeletal muscle cells were performed only in primary cells obtained from obese humans. We chose to perform these lipid incubation experiments in cells derived from obese subjects because obese human skeletal muscle is regularly exposed to high circulating fatty acid availability *in vivo* [9, 11], and we wanted to mimic this effect *in vitro*. However, the insulin resistant phenotype is often found to be retained in primary skeletal muscle cells derived from obese humans [26], which might help explain why the reduction in insulin signaling with our lipid incubations did not reach statistical significance in the primary muscle cells from our obese subjects.

Overall, our findings indicate that compared with the profound impairment in insulin signaling when cultured myotubes were incubated with palmitate, exposure to physiologic mixtures of fatty acids only modestly blunted insulin-stimulated Akt phosphorylation, even when the mixture contained a very high proportion of saturated fatty acids (60%). This may be explained in part by a selective partitioning of fatty acids into neutral lipid (i.e., TAG) when muscle cells were exposed to physiologic lipid mixtures. Our experiments in human primary muscle cells suggest that these findings translate to muscle from obese humans. Future studies aimed at elucidating the mechanism(s) underlying intracellular partitioning of fatty acids may yield insight into promising targets for the treatment of obesity-related insulin resistance.

## Supporting Information

S1 Fig Basal and insulin signaling in C2C12 muscle cells. Muscle cells were incubated with PALM (open), NORM (filled), or HSFA (hatched). Basal (i.e. non-insulin stimulated) (A) pAkt^Thr308^/Akt, and insulin-stimulated (B) pGSK3β^Ser9^/GSK3β and (C) pAS160^Thr642^/AS160. Data are expressed relative to a no fatty acid (0mM) condition. Representative blots are inset above each figure panel. #*P*<0.05 for a main effect of treatment dose *vs*. 0mM, ##*P*<0.05 for a main effect of NORM *vs*. PALM and HSFA. GSK, glycogen synthase kinase; AS160, Akt substrate of 160 kD; AU, arbitrary units.
(TIFF)

S1 Table Fatty acid composition of cellular diacylglycerol. Values are means for n = 3 expressed as a percentage of the total triacylglycerol fatty acid pool. The composition of the different fatty acid treatments in the incubation media is provided in the italicized rows.
(DOCX)

S2 Table Fatty acid composition of cellular triacylglycerol. Values are means for n = 3 expressed as a percentage of the total triacylglycerol fatty acid pool. The composition of the different fatty acid treatments in the incubation media is provided in the italicized rows.
(DOCX)

S3 Table Individual data for pAkt^Thr308^/Akt in C2C12 muscle cells. (DOCX)

S4 Table Individual data for DAG in C2C12 muscle cells. (DOCX)

S5 Table Individual data for TAG in C2C12 muscle cells. (DOCX)

S6 Table Individual data for GPAT1 in C2C12 muscle cells. (DOCX)

S7 Table Individual data for DGAT1 in C2C12 muscle cells. (DOCX)

S8 Table Individual data for ATGL in C2C12 muscle cells. (DOCX)

S9 Table Individual data for CGI58 in C2C12 muscle cells. (DOCX)

S10 Table Individual data for HSL in C2C12 muscle cells. (DOCX)

S11 Table Individual data for delta pAkt^Thr308^/Akt in human primary skeletal muscle cells. (DOCX)

S12 Table Individual data for DAG in human primary skeletal muscle cells. (DOCX)

S13 Table Individual data for TAG in human primary skeletal muscle cells. (DOCX)
