# Supplementary figures and images for: Lipid Mixtures Containing a Very High Proportion of Saturated Fatty Acids Only Modestly Impair Insulin Signaling in Cultured Muscle Cells
Source: PLoS One. 2015 Mar 20;10(3):e0120871. doi: 10.1371/journal.pone.0120871 (PMC4368748; doi:10.1371/journal.pone.0120871)

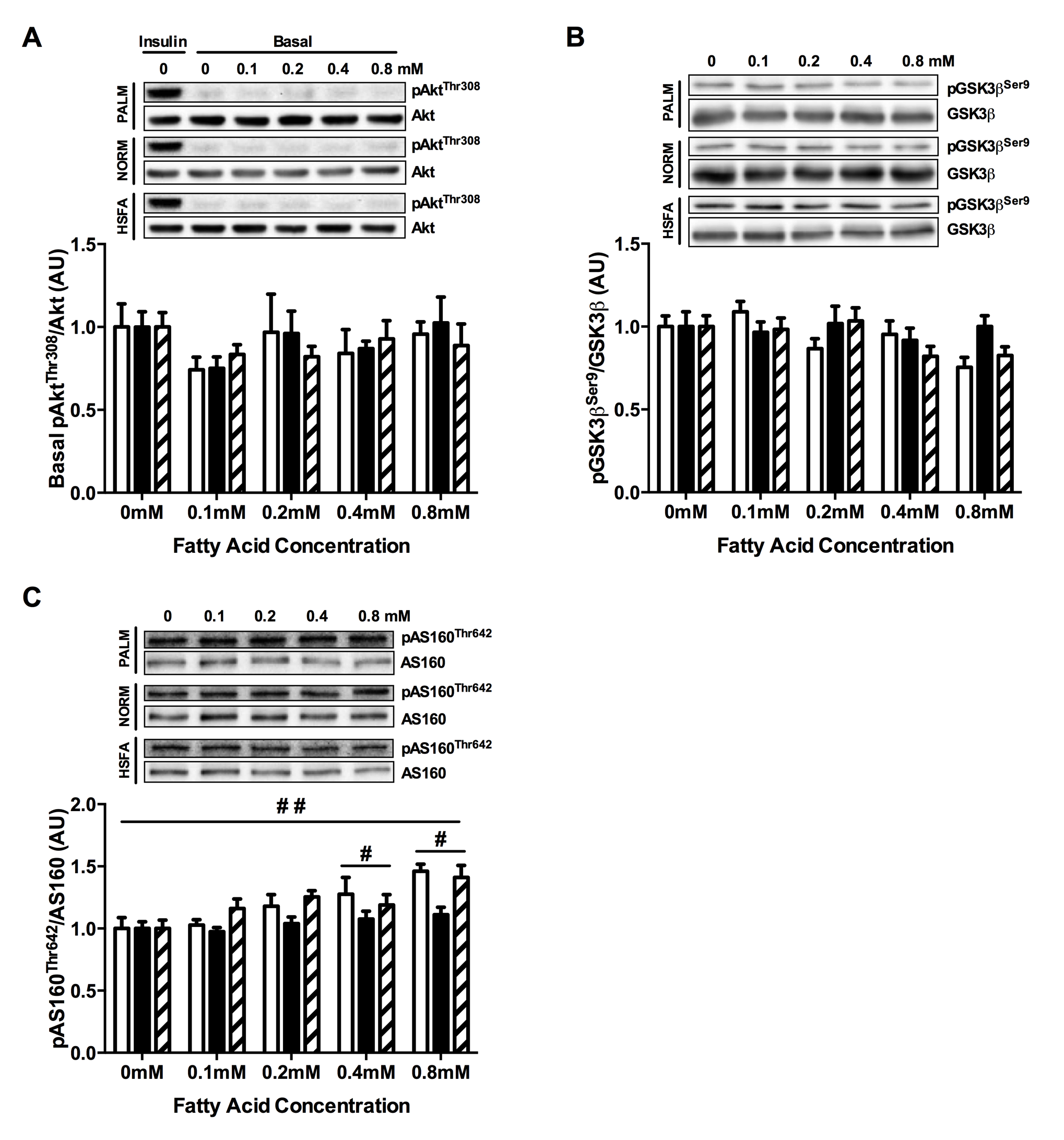

Supplement: S1 Fig — Muscle cells were incubated with PALM (open), NORM (filled), or HSFA (hatched). Basal (i.e. non-insulin stimulated) (A) pAktThr308/Akt, and insulin-stimulated (B) pGSK3βSer9/GSK3β and (C) pAS160Thr642/AS160. Data are expressed relative to a no fatty acid (0mM) condition. Representative blots are inset above each figure panel. #P<0.05 for a main effect of treatment dose vs. 0mM, ##P<0.05 for a main effect of NORM vs. PALM and HSFA. GSK, glycogen synthase kinase; AS160, Akt substrate of 160 kD; AU, arbitrary units. (TIFF) [file pone.0120871.s001.tiff]
