# Supplementary material for: Lipid Mixtures Containing a Very High Proportion of Saturated Fatty Acids Only Modestly Impair Insulin Signaling in Cultured Muscle Cells
Source: PLoS One. 2015 Mar 20;10(3):e0120871. doi: 10.1371/journal.pone.0120871 (PMC4368748; doi:10.1371/journal.pone.0120871)
Supplement: S1 Table — Values are means for n = 3 expressed as a percentage of the total triacylglycerol fatty acid pool. The composition of the different fatty acid treatments in the incubation media is provided in the italicized rows. (DOCX) [file pone.0120871.s002.docx]

| **Table S1. Fatty acid composition of cellular diacylglycerol (% of the total diacylglycerol fatty acid pool)** | | | | | | |
| --- | --- | --- | --- | --- | --- | --- |
| **Treatment Type** | **mM** | **C16:0** | **C18:0** | **C16:1** | **C18:1** | **C18:2** |
| No fatty acid  (Control) | 0 | 24% | 3% | 17% | 49% | 4% |
| *PALM incubation media* | | *100%* | *0%* | *0%* | *0%* | *0%* |
| PALM | 0.1 | 42% | 22% | 9% | 25% | 1% |
| PALM | 0.2 | 50% | 23% | 8% | 17% | 0% |
| PALM | 0.4 | 54% | 18% | 9% | 17% | 1% |
| PALM | 0.8 | 76% | 12% | 5% | 7% | 0% |
| *NORM incubation media* | | *25%* | *15%* | *5%* | *30%* | *25%* |
| NORM | 0.1 | 22% | 24% | 4% | 37% | 11% |
| NORM | 0.2 | 21% | 25% | 6% | 34% | 13% |
| NORM | 0.4 | 26% | 28% | 3% | 30% | 13% |
| NORM | 0.8 | 24% | 26% | 3% | 29% | 17% |
| *HSFA incubation media* | | *35%* | *25%* | *5%* | *20%* | *15%* |
| HSFA | 0.1 | 20% | 22% | 13% | 43% | 2% |
| HSFA | 0.2 | 23% | 18% | 6% | 49% | 3% |
| HSFA | 0.4 | 24% | 13% | 9% | 45% | 9% |
| HSFA | 0.8 | 32% | 14% | 8% | 36% | 10% |
| Values are means for n=3 expressed as a percentage of the total diacylglycerol fatty acid pool. The composition of the different fatty acid treatments in the incubation media is provided in the italicized rows. | | | | | | |
