# Supplementary material for: Lipid Mixtures Containing a Very High Proportion of Saturated Fatty Acids Only Modestly Impair Insulin Signaling in Cultured Muscle Cells
Source: PLoS One. 2015 Mar 20;10(3):e0120871. doi: 10.1371/journal.pone.0120871 (PMC4368748; doi:10.1371/journal.pone.0120871)
Supplement: S2 Table — Values are means for n = 3 expressed as a percentage of the total triacylglycerol fatty acid pool. The composition of the different fatty acid treatments in the incubation media is provided in the italicized rows. (DOCX) [file pone.0120871.s003.docx]

| **Table S2. Fatty acid composition of cellular triacylglycerol (% of the total triacylglycerol fatty acid pool)** | | | | | | |
| --- | --- | --- | --- | --- | --- | --- |
| **Treatment Type** | **mM** | **C16:0** | **C18:0** | **C16:1** | **C18:1** | **C18:2** |
| No fatty acid  (Control) | 0 | 24% | 3% | 17% | 49% | 4% |
| *PALM incubation media* | | *100%* | *0%* | *0%* | *0%* | *0%* |
| PALM | 0.1 | 50% | 1% | 14% | 31% | 2% |
| PALM | 0.2 | 62% | 2% | 11% | 22% | 2% |
| PALM | 0.4 | 72% | 1% | 9% | 16% | 1% |
| PALM | 0.8 | 83% | 2% | 5% | 8% | 1% |
| *NORM incubation media* | | *25%* | *15%* | *5%* | *30%* | *25%* |
| NORM | 0.1 | 22% | 4% | 10% | 51% | 12% |
| NORM | 0.2 | 21% | 5% | 7% | 49% | 17% |
| NORM | 0.4 | 23% | 7% | 5% | 43% | 22% |
| NORM | 0.8 | 24% | 8% | 3% | 39% | 25% |
| *HSFA incubation media* | | *35%* | *25%* | *5%* | *20%* | *15%* |
| HSFA | 0.1 | 28% | 6% | 12% | 46% | 7% |
| HSFA | 0.2 | 31% | 7% | 9% | 44% | 8% |
| HSFA | 0.4 | 34% | 10% | 6% | 40% | 10% |
| HSFA | 0.8 | 35% | 14% | 4% | 35% | 12% |
| Values are means for n=3 expressed as a percentage of the total triacylglycerol fatty acid pool. The composition of the different fatty acid treatments in the incubation media is provided in the italicized rows. | | | | | | |
