# Supplementary material for: Lipid Mixtures Containing a Very High Proportion of Saturated Fatty Acids Only Modestly Impair Insulin Signaling in Cultured Muscle Cells
Source: PLoS One. 2015 Mar 20;10(3):e0120871. doi: 10.1371/journal.pone.0120871 (PMC4368748; doi:10.1371/journal.pone.0120871)
Supplement: S3 Table — (DOCX) [file pone.0120871.s004.docx]

| **Table S3. Individual data for pAkt^Thr308^/Akt in C2C12 muscle cells** | | | | |
| --- | --- | --- | --- | --- |
| ***PALM Treatment*** | | | | |
| **0 mM** | **0.1 mM** | **0.2 mM** | **0.4 mM** | **0.8 mM** |
| 0.853 | 0.816 | 0.449 | 0.347 | 0.351 |
| 0.958 | 0.579 | 0.445 | 0.519 | 0.316 |
| 0.865 | 0.627 | 0.520 | 0.436 | 0.368 |
| 0.836 | 0.652 | 0.558 | 0.352 | 0.419 |
| 1.055 | 0.565 | 0.369 | 0.427 | 0.402 |
| 0.891 | 0.642 | 0.566 | 0.383 | 0.334 |
| 1.309 | 0.540 | 0.346 | 0.293 | 0.329 |
| 1.173 | 0.405 | 0.319 | 0.411 | 0.509 |
| 1.060 | 0.555 | 0.381 | 0.433 | 0.388 |
| ***NORM Treatment*** | | | | |
| **0 mM** | **0.1 mM** | **0.2 mM** | **0.4 mM** | **0.8 mM** |
| 0.986 | 0.556 | 0.712 | 0.862 | 1.009 |
| 1.108 | 0.666 | 0.829 | 0.673 | 0.848 |
| 0.749 | 0.900 | 0.571 | 0.805 | 1.101 |
| 0.978 | 0.858 | 0.611 | 0.610 | 1.068 |
| 0.996 | 0.786 | 0.650 | 0.759 | 0.935 |
| 0.929 | 0.842 | 0.854 | 0.703 | 0.798 |
| 1.171 | 0.811 | 0.670 | 0.714 | 0.760 |
| 1.265 | 0.821 | 0.591 | 0.653 | 0.796 |
| 0.818 | 0.939 | 0.648 | 0.391 | 1.329 |
| ***HSFA Treatment*** | | | | |
| **0 mM** | **0.1 mM** | **0.2 mM** | **0.4 mM** | **0.8 mM** |
| 1.359 | 0.706 | 0.645 | 0.513 | 0.682 |
| 0.938 | 1.005 | 0.688 | 0.586 | 0.688 |
| 0.872 | 0.801 | 0.692 | 0.793 | 0.748 |
| 1.210 | 0.780 | 0.661 | 0.679 | 0.576 |
| 1.312 | 0.683 | 0.602 | 0.534 | 0.775 |
| 0.983 | 0.519 | 0.662 | 0.834 | 0.908 |
| 0.855 | 0.739 | 0.698 | 1.070 | 0.544 |
| 0.511 | 0.549 | 0.637 | 1.221 | 0.987 |
| 0.958 | 0.854 | 1.121 | 0.595 | 0.377 |
