# Supplementary material for: Lipid Mixtures Containing a Very High Proportion of Saturated Fatty Acids Only Modestly Impair Insulin Signaling in Cultured Muscle Cells
Source: PLoS One. 2015 Mar 20;10(3):e0120871. doi: 10.1371/journal.pone.0120871 (PMC4368748; doi:10.1371/journal.pone.0120871)
Supplement: S4 Table — (DOCX) [file pone.0120871.s005.docx]

| **Table S4. Individual data for DAG in C2C12 muscle cells** | | | | |
| --- | --- | --- | --- | --- |
| ***PALM Treatment*** | | | | |
| **0 mM** | **0.1 mM** | **0.2 mM** | **0.4 mM** | **0.8 mM** |
| 1.15 | 1.77 | 1.81 | 2.55 | 4.24 |
| 1.39 | 1.95 | 2.37 | 2.90 | 5.50 |
| 0.65 | 0.83 | 0.95 | 1.43 | 3.31 |
| 1.32 | 1.98 | 2.39 | 3.59 | 6.65 |
| 0.49 |  | 1.42 | 1.69 | 4.61 |
| ***NORM Treatment*** | | | | |
| **0 mM** | **0.1 mM** | **0.2 mM** | **0.4 mM** | **0.8 mM** |
| 1.15 | 1.92 | 1.86 | 1.57 | 1.90 |
| 1.39 | 1.96 | 1.81 | 2.37 | 2.78 |
| 0.65 | 1.21 | 1.29 | 1.31 | 1.77 |
| 1.32 | 1.04 | 1.07 | 1.69 | 1.63 |
| 0.49 | 0.56 | 0.76 |  | 0.88 |
| ***HSFA Treatment*** | | | | |
| **0 mM** | **0.1 mM** | **0.2 mM** | **0.4 mM** | **0.8 mM** |
| 1.15 | 0.87 | 0.86 | 1.39 | 1.28 |
| 1.39 | 0.68 | 0.48 | 0.79 | 1.47 |
| 0.65 | 0.86 | 1.17 | 1.38 | 1.99 |
| 1.32 | 1.32 | 1.46 | 1.17 | 1.68 |
| 0.49 | 0.82 | 0.85 | 0.76 | 0.85 |
