# Supplementary material for: Lipid Mixtures Containing a Very High Proportion of Saturated Fatty Acids Only Modestly Impair Insulin Signaling in Cultured Muscle Cells
Source: PLoS One. 2015 Mar 20;10(3):e0120871. doi: 10.1371/journal.pone.0120871 (PMC4368748; doi:10.1371/journal.pone.0120871)
Supplement: S5 Table — (DOCX) [file pone.0120871.s006.docx]

| **Table S5. Individual data for TAG in C2C12 muscle cells** | | | | |
| --- | --- | --- | --- | --- |
| ***PALM Treatment*** | | | | |
| **0 mM** | **0.1 mM** | **0.2 mM** | **0.4 mM** | **0.8 mM** |
| 0.95 | 2.48 | 2.77 | 4.12 | 4.91 |
| 0.91 | 1.74 | 2.29 | 2.59 | 5.00 |
| 1.14 | 2.25 | 2.77 | 4.02 | 5.93 |
| ***NORM Treatment*** | | | | |
| **0 mM** | **0.1 mM** | **0.2 mM** | **0.4 mM** | **0.8 mM** |
| 0.95 | 3.27 | 4.11 | 5.49 | 9.04 |
| 0.91 | 3.23 | 3.39 | 5.87 | 11.58 |
| 1.14 | 2.77 | 3.68 | 6.17 | 10.90 |
| ***HSFA Treatment*** | | | | |
| **0 mM** | **0.1 mM** | **0.2 mM** | **0.4 mM** | **0.8 mM** |
| 0.95 | 2.25 | 3.07 | 6.78 | 7.95 |
| 0.91 | 3.16 | 3.76 | 5.99 | 8.93 |
| 1.14 | 2.19 | 3.69 | 5.44 | 12.21 |
