# Supplementary material for: Lipid Mixtures Containing a Very High Proportion of Saturated Fatty Acids Only Modestly Impair Insulin Signaling in Cultured Muscle Cells
Source: PLoS One. 2015 Mar 20;10(3):e0120871. doi: 10.1371/journal.pone.0120871 (PMC4368748; doi:10.1371/journal.pone.0120871)
Supplement: S6 Table — (DOCX) [file pone.0120871.s007.docx]

| **Table S6. Individual data for GPAT1 in C2C12 muscle cells** | | | | |
| --- | --- | --- | --- | --- |
| ***PALM Treatment*** | | | | |
| **0 mM** | **0.1 mM** | **0.2 mM** | **0.4 mM** | **0.8 mM** |
| 0.579 | 0.578 | 1.168 | 1.109 | 1.487 |
| 1.595 | 1.266 | 1.072 | 1.576 | 1.018 |
| 1.048 | 1.049 | 0.820 | 0.834 | 1.154 |
| 0.778 | 0.938 | 0.710 | 0.780 | 0.487 |
| ***NORM Treatment*** | | | | |
| **0 mM** | **0.1 mM** | **0.2 mM** | **0.4 mM** | **0.8 mM** |
| 0.976 | 0.645 | 1.260 | 1.468 | 1.199 |
| 0.886 | 0.883 | 1.128 | 0.900 | 0.850 |
| 1.396 | 1.479 | 1.446 | 1.172 | 1.261 |
| 0.743 | 0.771 | 0.820 | 0.895 | 1.526 |
| ***HSFA Treatment*** | | | | |
| **0 mM** | **0.1 mM** | **0.2 mM** | **0.4 mM** | **0.8 mM** |
| 0.597 | 0.895 | 0.900 | 0.769 | 0.954 |
| 1.160 | 0.843 | 0.766 | 0.879 | 0.695 |
| 0.683 | 0.617 | 0.731 | 0.564 | 0.458 |
| 1.560 | 1.062 | 1.348 | 1.001 | 1.143 |
