# Supplementary material for: Lipid Mixtures Containing a Very High Proportion of Saturated Fatty Acids Only Modestly Impair Insulin Signaling in Cultured Muscle Cells
Source: PLoS One. 2015 Mar 20;10(3):e0120871. doi: 10.1371/journal.pone.0120871 (PMC4368748; doi:10.1371/journal.pone.0120871)
Supplement: S7 Table — (DOCX) [file pone.0120871.s008.docx]

| **Table S7. Individual data for DGAT1 in C2C12 muscle cells** | | | | |
| --- | --- | --- | --- | --- |
| ***PALM Treatment*** | | | | |
| **0 mM** | **0.1 mM** | **0.2 mM** | **0.4 mM** | **0.8 mM** |
| 1.071 | 1.278 | 0.890 | 0.677 | 0.795 |
| 1.758 | 1.911 | 1.344 | 1.050 | 0.959 |
| 0.790 | 1.135 | 0.919 | 0.853 | 1.093 |
| 0.559 | 0.504 | 0.379 | 0.415 | 0.455 |
| 1.027 | 1.219 | 0.132 | 1.023 | 1.028 |
| 0.796 | 0.909 | 0.802 | 0.990 | 0.979 |
| ***NORM Treatment*** | | | | |
| **0 mM** | **0.1 mM** | **0.2 mM** | **0.4 mM** | **0.8 mM** |
| 0.759 | 0.736 | 0.539 | 0.516 | 0.570 |
| 0.584 | 0.525 | 0.588 | 0.619 | 0.660 |
| 1.150 | 0.800 | 0.934 | 0.702 | 0.776 |
| 0.843 | 0.734 | 0.495 | 0.657 | 0.805 |
| 1.922 | 1.245 | 1.130 | 1.311 | 0.960 |
| 0.743 | 0.499 | 0.575 | 0.748 | 0.558 |
| ***HSFA Treatment*** | | | | |
| **0 mM** | **0.1 mM** | **0.2 mM** | **0.4 mM** | **0.8 mM** |
| 1.147 | 0.785 | 1.345 | 1.277 | 1.253 |
| 1.241 | 1.579 | 1.161 | 0.970 | 0.930 |
| 1.144 | 1.464 | 1.268 | 1.117 | 1.384 |
| 0.932 | 0.880 | 0.982 | 1.138 | 0.969 |
| 0.816 | 1.191 | 1.392 | 1.314 | 1.681 |
| 0.719 | 1.004 | 0.971 | 1.248 | 1.025 |
