# Supplementary material for: Lipid Mixtures Containing a Very High Proportion of Saturated Fatty Acids Only Modestly Impair Insulin Signaling in Cultured Muscle Cells
Source: PLoS One. 2015 Mar 20;10(3):e0120871. doi: 10.1371/journal.pone.0120871 (PMC4368748; doi:10.1371/journal.pone.0120871)
Supplement: S8 Table — (DOCX) [file pone.0120871.s009.docx]

| **Table S8. Individual data for ATGL in C2C12 muscle cells** | | | | |
| --- | --- | --- | --- | --- |
| ***PALM Treatment*** | | | | |
| **0 mM** | **0.1 mM** | **0.2 mM** | **0.4 mM** | **0.8 mM** |
| 0.749 | 0.893 | 1.554 | 1.772 | 2.589 |
| 1.116 | 1.366 | 1.755 | 2.116 | 1.185 |
| 1.049 | 0.750 | 1.136 | 1.410 | 2.224 |
| 0.660 | 1.740 | 2.742 | 4.968 | 6.006 |
| 0.871 | 1.058 | 1.586 | 1.189 | 1.318 |
| 1.554 | 2.001 | 2.350 | 2.508 | 2.252 |
| ***NORM Treatment*** | | | | |
| **0 mM** | **0.1 mM** | **0.2 mM** | **0.4 mM** | **0.8 mM** |
| 1.107 | 1.529 | 1.483 | 3.455 | 3.234 |
| 1.057 | 1.270 | 2.409 | 2.111 | 4.001 |
| 0.597 | 0.812 | 0.802 | 1.595 | 1.644 |
| 0.614 | 0.727 | 0.779 | 2.087 | 3.350 |
| 1.631 | 1.721 | 2.001 | 3.228 | 4.570 |
| 0.994 | 0.749 | 1.171 | 1.529 | 2.171 |
| ***HSFA Treatment*** | | | | |
| **0 mM** | **0.1 mM** | **0.2 mM** | **0.4 mM** | **0.8 mM** |
| 1.374 | 1.786 | 4.011 | 0.498 | 5.466 |
| 1.921 | 2.251 | 3.385 | 4.715 | 6.431 |
| 0.274 | 0.369 | 0.574 | 0.723 | 0.821 |
| 1.053 | 1.305 | 2.153 | 2.252 | 3.277 |
| 0.772 | 1.225 | 2.095 | 3.233 | 4.282 |
| 0.606 | 0.894 | 1.994 | 2.863 | 2.917 |
