# Supplementary material for: Lipid Mixtures Containing a Very High Proportion of Saturated Fatty Acids Only Modestly Impair Insulin Signaling in Cultured Muscle Cells
Source: PLoS One. 2015 Mar 20;10(3):e0120871. doi: 10.1371/journal.pone.0120871 (PMC4368748; doi:10.1371/journal.pone.0120871)
Supplement: S9 Table — (DOCX) [file pone.0120871.s010.docx]

| **Table S9. Individual data for CGI58 in C2C12 muscle cells** | | | | |
| --- | --- | --- | --- | --- |
| ***PALM Treatment*** | | | | |
| **0 mM** | **0.1 mM** | **0.2 mM** | **0.4 mM** | **0.8 mM** |
| 1.041 | 1.084 | 1.617 | 1.096 | 1.621 |
| 0.908 | 0.792 | 1.043 | 0.809 | 0.959 |
| 0.799 | 0.731 | 0.608 | 0.824 | 0.816 |
| 1.252 | 1.159 | 1.037 | 1.187 | 0.777 |
| ***NORM Treatment*** | | | | |
| **0 mM** | **0.1 mM** | **0.2 mM** | **0.4 mM** | **0.8 mM** |
| 0.969 | 1.018 | 1.021 | 0.959 | 0.968 |
| 0.817 | 0.986 | 0.782 | 0.714 | 1.018 |
| 1.042 | 0.796 | 1.074 | 1.074 | 1.277 |
| 1.172 | 0.963 | 0.858 | 1.198 | 1.006 |
| ***HSFA Treatment*** | | | | |
| **0 mM** | **0.1 mM** | **0.2 mM** | **0.4 mM** | **0.8 mM** |
| 0.846 | 1.130 | 1.178 | 0.911 | 1.272 |
| 1.332 | 1.718 | 1.259 | 1.411 | 0.921 |
| 1.069 | 1.086 | 0.130 | 0.941 | 0.953 |
| 0.753 | 0.992 | 1.034 | 0.695 | 1.141 |
