# Supplementary material for: Lipid Mixtures Containing a Very High Proportion of Saturated Fatty Acids Only Modestly Impair Insulin Signaling in Cultured Muscle Cells
Source: PLoS One. 2015 Mar 20;10(3):e0120871. doi: 10.1371/journal.pone.0120871 (PMC4368748; doi:10.1371/journal.pone.0120871)
Supplement: S10 Table — (DOCX) [file pone.0120871.s011.docx]

| **Table S10. Individual data for HSL in C2C12 muscle cells** | | | | |
| --- | --- | --- | --- | --- |
| ***PALM Treatment*** | | | | |
| **0 mM** | **0.1 mM** | **0.2 mM** | **0.4 mM** | **0.8 mM** |
| 0.919 | 1.004 | 1.356 | 1.168 | 0.852 |
| 0.584 | 1.107 | 1.271 | 0.867 | 0.481 |
| 0.979 | 0.867 | 0.987 | 1.090 | 0.755 |
| 1.249 | 1.294 | 1.033 | 1.362 | 1.260 |
| 1.056 | 2.384 | 1.862 | 1.192 | 1.328 |
| 1.215 | 1.124 | 1.181 | 1.249 | 1.022 |
| ***NORM Treatment*** | | | | |
| **0 mM** | **0.1 mM** | **0.2 mM** | **0.4 mM** | **0.8 mM** |
| 0.931 | 1.195 | 1.086 | 1.055 | 1.102 |
| 0.931 | 0.853 | 0.838 | 0.690 | 0.884 |
| 0.771 | 0.918 | 0.935 | 0.935 | 0.886 |
| 0.804 | 0.696 | 0.781 | 0.747 | 0.968 |
| 1.166 | 1.184 | 1.203 | 1.554 | 1.554 |
| 1.398 | 1.289 | 1.325 | 1.489 | 1.344 |
| ***HSFA Treatment*** | | | | |
| **0 mM** | **0.1 mM** | **0.2 mM** | **0.4 mM** | **0.8 mM** |
| 0.783 | 1.261 | 1.121 | 1.228 | 1.615 |
| 0.906 | 1.269 | 1.401 | 1.327 | 0.849 |
| 0.791 | 1.046 | 0.808 | 0.840 | 0.923 |
| 1.104 | 1.363 | 1.046 | 1.257 | 0.999 |
| 1.260 | 1.386 | 1.433 | 1.328 | 1.093 |
| 1.155 | 1.307 | 1.079 | 1.395 | 1.362 |
