# Supplementary material for: Lipid Mixtures Containing a Very High Proportion of Saturated Fatty Acids Only Modestly Impair Insulin Signaling in Cultured Muscle Cells
Source: PLoS One. 2015 Mar 20;10(3):e0120871. doi: 10.1371/journal.pone.0120871 (PMC4368748; doi:10.1371/journal.pone.0120871)
Supplement: S11 Table — (DOCX) [file pone.0120871.s012.docx]

| **Table S11. Individual data for delta pAkt^Thr308^/Akt in human primary skeletal muscle cells** | | | |
| --- | --- | --- | --- |
| **CON** | **PALM** | **NORM** | **HSFA** |
| 1.375 | 0.328 | 0.194 | 0.439 |
| 0.064 | 0.250 | 0.393 |  |
| -0.047 | 0.007 | 0.065 | 0.161 |
| 0.425 | 0.084 | 0.194 | 0.618 |
| 1.471 | 0.243 | 0.818 | 0.706 |
| 2.712 | 0.692 | 1.431 | 1.474 |
