# Supplementary material for: Lipid Mixtures Containing a Very High Proportion of Saturated Fatty Acids Only Modestly Impair Insulin Signaling in Cultured Muscle Cells
Source: PLoS One. 2015 Mar 20;10(3):e0120871. doi: 10.1371/journal.pone.0120871 (PMC4368748; doi:10.1371/journal.pone.0120871)
Supplement: S12 Table — (DOCX) [file pone.0120871.s013.docx]

| **Table S12. Individual data for DAG in human primary skeletal muscle cells** | | | |
| --- | --- | --- | --- |
| **CON** | **PALM** | **NORM** | **HSFA** |
| 1.50 | 2.40 | 3.15 | 4.17 |
| 1.30 | 2.41 | 0.92 | 3.17 |
| 0.20 | 1.64 | 1.17 | 3.22 |
