# Supplementary material for: Lipid Mixtures Containing a Very High Proportion of Saturated Fatty Acids Only Modestly Impair Insulin Signaling in Cultured Muscle Cells
Source: PLoS One. 2015 Mar 20;10(3):e0120871. doi: 10.1371/journal.pone.0120871 (PMC4368748; doi:10.1371/journal.pone.0120871)
Supplement: S13 Table — (DOCX) [file pone.0120871.s014.docx]

| **Table S13. Individual data for TAG in human primary skeletal muscle cells** | | | |
| --- | --- | --- | --- |
| **CON** | **PALM** | **NORM** | **HSFA** |
| 1.77 | 8.81 | 8.85 | 7.91 |
| 0.82 | 11.82 | 10.00 | 8.05 |
| 0.41 | 7.42 | 5.84 | 4.78 |
